# Biomedical Application of Nanogels: From Cancer to Wound Healing

**DOI:** 10.3390/molecules30102144

**Published:** 2025-05-13

**Authors:** Mohammad Zafaryab, Komal Vig

**Affiliations:** Department of Biological Sciences, Alabama State University, Montgomery, AL 36104, USA; mzafaryab@alasu.edu

**Keywords:** nanogel, wound healing, anticancer, hydrogel

## Abstract

Nanogels are polymer-based, crosslinked hydrogel particles on the nanometer scale. Nanogels developed from synthetic and natural polymers have gathered a great deal of attention in industry and scientific society due to having an increased surface area, softness, flexibility, absorption, and drug loading ability, as well as their mimicking the environment of a tissue. Nanogels having biocompatibility, nontoxic and biodegradable properties with exceptional design, fabrication, and coating facilities may be used for a variety of different biomedical applications, such as drug delivery and therapy, tissue engineering, and bioimaging. Nanogels fabricated by chemical crosslinking and physical self-assembly displayed the ability to encapsulate therapeutics, including hydrophobic, hydrophilic, and small molecules, proteins, peptides, RNA and DNA sequences, and even ultrasmall nanoparticles within their three-dimensional polymer networks. One of the many drug delivery methods being investigated as a practical option for targeted delivery of drugs for cancer treatment is nanogels. The delivery of DNA and anticancer drugs like doxorubicin, epirubicin, and paclitaxel has been eased by polymeric nanogels. Stimuli-responsive PEGylated nanogels have been reported as smart nanomedicines for cancer diagnostics and therapy. Another promising biomedical application of nanogels is wound healing. Wounds are injuries to living tissue caused by a cut, blow, or other impact. There are numerous nanogels having different polymer compositions that have been reported to enhance the wound healing process, such as hyaluronan, poly-L-lysine, and berberine. When antimicrobial resistance is present, wound healing becomes a complicated process. Researchers are looking for novel alternative approaches, as foreign microorganisms in wounds are becoming resistant to antibiotics. Silver nanogels have been reported as a popular antimicrobial choice, as silver has been used as an antimicrobial throughout a prolonged period. Lignin-incorporated nanogels and lidocaine nanogels have also been reported as an antioxidant wound-dressing material that can aid in wound healing. In this review, we will summarize recent progress in biomedical applications for various nanogels, with a prime focus on cancer and wound healing.

## 1. Background

The term “nanogel” was originally used when the polymers: poly (ethylene glycol) and poly (ethyleneimine) were chemically crosslinked to generate hydrophilic polymer networks for antisense oligonucleotide delivery [1]. Nanogels vary in size from 20 to 200 nm. Their size allows them to elude renal clearance and prolong the half-life of serum clearance [2].

Nanogels are the three-dimensional version of hydrogels, within the nanoscale size range nanogels are composed of networks of crosslinked swellable polymers with a high water-holding ability and do not dissolve in aqueous conditions. A range of naturally occurring polymers, synthetic polymers, or a combination of both can be used to create nanogels. The softness, porosity, size, charge, amphiphilicity, and degradability of nanogels can all be adjusted by varying their chemical makeup. Nanogels are mostly spherical in nature, but advancements in synthetic methods allow for the creation of nanogels in a wide range of shapes [1,3,4]. One of the most distinctive features of nanogels is their swelling tendency, which is caused by water being absorbed by the polymer. This characteristic makes the material primarily hydrophilic, meaning that it can absorb large volumes of water or biological fluids without losing its structural integrity [5]. Nanogels actively take part in the delivery process, in addition to protecting the cargo from degradation and removal. Special qualities of nanogel like swelling, softness, and stimuli-responsive behavior aid in setting up a controlled and triggered reaction at the target site [6,7]. Their structure allows addition of a variety of guest molecules, from inorganic nanoparticles to biomacromolecules like proteins and DNA, without affecting their gel-like properties [8,9]. Nanogels are perfect for a variety of drug delivery applications because of their special ability to encapsulate several bioactive components in a single carrier. Through the specific choice of polymers and architectural adaptability that permits the inclusion of various molecules while keeping their gel-like behavior, nanogels in drug delivery not only protect payloads from degradation and premature release but also actively engage in the delivery process [10].

## 2. Classification of Nanogels

**Figure 1:**
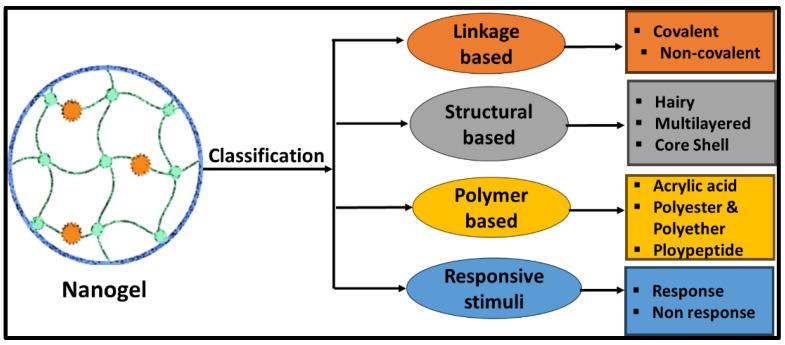
Classification of nanogels based on structure, linkage, polymer composition, and stimuli responsiveness.

Nanogels are broadly classified into four groups based on linkage, the structure of the polymer, polymer composition, and stimuli responsiveness (Figure 1).

### 2.1. Linkage-Based Nanogel

A range of natural, synthetic, or a combination of both polymers can be used to create nanogels. Polymers can be physically or chemically crosslinked with noncovalent bonds through hydrogen bonding, electrostatic interactions, and hydrophobic interactions [11]. Physical interactions that self-assemble and do not require crosslinking agents are called noncovalent linkages. The physical self-assembly of interactive polymers are triggered by the controlled aggregation through noncovalent or lower interactions such as hydrogen bonding, ionic contacts, van der Waals forces, hydrophilic–hydrophilic, and hydrophobic–hydrophobic interactions [12]. Ionic-based nanogels allow for targeted drug delivery, gene therapy, and imaging due to their ability to adjust size, shape, modulate surface charges, and response to environmental conditions including pH and ionic strength. Their capacity to encapsulate hydrophobic and hydrophilic chemicals, as well as their biocompatibility and biodegradability, increase their potential for use in cancer treatment, wound healing, and controlled drug release. The ionic crosslinking method also makes biologics such as proteins, enzymes, and nucleic acid suitable for tissue engineering and gene delivery by enabling their prolonged release and protection [13,14,15].

Nanoscale polymer networks created by covalent bonding are known as covalent crosslinked nanogels. These materials have special qualities like excellent stability, large surface area, water dispersibility, and biocompatibility, which make them perfect for biomedical applications [16]. These nanogels make it possible to release therapeutic drug in a controlled and prolonged manner due to their high drug loading capacity, adaptability for targeted delivery, and stimuli-responsive properties (pH or temperature sensitivity) [17]. The pliable nature of these nanogels makes them useful for wound healing, gene therapy, tissue engineering, drug delivery, and cancer treatment. These nanogels are appropriate for delivering drugs, proteins, and nucleic acids to certain areas, enhancing therapeutic efficiency and reducing side effects because of their capacity to encapsulate and safeguard bioactive components while guaranteeing efficient release [18].

### 2.2. Structural-Based Nanogels

Nanogels can further be categorized based on their structural features.

#### 2.2.1. Hollow Nanogels

Poly(N-isopropylacrylamide) (PNIPAM) was applied to the surface-modified silica colloidal particles by Zha et al. using the precipitation polymerization procedure to produce temperature-responsive nanogels with a hollow structure, silica particles were then removed [19]. By altering the template size, hollow nanogels size can be regulated. It is also feasible to change the thickness of the hollow nanogel shell by changing the mass ratio of monomer and template. A volume phase change occurs in the hollow nanogels at about 32 °C. Upon external cues, permeability of hollow nanogel shells can be altered, which eases the regulated release of guest molecules. Xing et al. developed pH/temperature-responsive hollow nanogels using a similar method [20]. Drugs can be added to the hollow nanogels by varying the temperature and pH of the surrounding fluid, which can control drug release rate [21].

#### 2.2.2. Multilayered Nanogels

These nanogels are composed of either one polymer or many layers of distinct polymers. Achieving high site specificity requires careful consideration while choosing the proper polymers. This delivery strategy is effective for hazardous or sensitive drugs because the polymer arrangement affects the drugs’ release profile. When addressing strong or low therapeutic effectiveness, multi-layered nanogels can be highly beneficial since they can be tailored to provide high site specificity. But their cost, scalability, yield, and the complexity make them difficult for clinical use. Moreover, peptides, oligopeptides, and nucleotides can be delivered with them. Taking advantage of its multi-layer structure, a new gellan pullulan nanogel was created by chemically crosslinking methylene blue out of the gel [22].

#### 2.2.3. Hairy Nanogels

Nanogels with thin projections on their surface that resemble hairs are known as hairy nanogels. These are made with macro-reversible addition fragmentation (RAFT) agents or controlled radical polymerization. The polymers that are subsequently covalently bonded to the core gel matrix give rise to these hairy projections [23]. A hairy nanogel is a core–shell structure, with linear polymeric chains in the shell that have a higher affinity for the dispersion medium and a nanosized core that exhibits the nano-effect [24,25].

#### 2.2.4. Core–Shell Nanogels

These systems have an outer shell that surrounds an inner core, which is typically made of metallic, bimetallic materials, carbon dots, or nanorods. The outer layer can be positioned by physical trapping or chemical bonding and can consist of organic structures or polymeric chains. Because of its adaptability, the core–shell nanogels can be tailored for several uses, including medicine administration for appropriate payload release or sensors for responsive behavior [26].

### 2.3. Polymer-Based Nanogels

Nanogels can be grouped according to the type of polymers that were utilized in their production. Natural, synthetic, or a combination of natural and synthetic polymers can be used to create nanogels [27]. Proteins are the primary natural polymers that are frequently employed, e.g., collagen, gelatin, fibrin, albumin, FBS and polysaccharides [28,29]. Other polymers included hyaluronic acid, pullulan, chitosan, chondroitin sulfate, agarose, alginate, cellulose, heparin, gellan gum, carragenan, and guar gum. Examples of synthetic polymers include PEG, polyglycolic derivatives, PEI, dendrimers, polypeptides, polyacrylates, polymethacrylates, poly (lactic acid) (PLA), poly(ε-caprolactone), and poly (lactic-co-glycolic acid) [30,31]. PEG, PEI, and dendrimers are often utilized in the fabrication of nanogels. PEG is most commonly added to the surface of nanoparticles (NPs) to increase colloidal stability, decrease uptake by the reticuloendothelial system (RES)/mononuclear phagocyte system (MPS), and prolong the circulation time because of its hydrophilic composition, which can produce the so-called “steric stabilization” effect [31,32,33].

### 2.4. Stimuli-Based Nanogels

External triggers are no longer necessary for nonresponsive nanogels to release drugs continuously at the target region because they swell through simple absorption upon encountering aqueous fluids [34]. However, responsive nanogels may swell or de-swell in response to changes in the following environmental parameters: electricity, pH, light, magnetic field, ultrasound, ion strength, and solvent composition [34].

## 3. Advantages and Shortcomings of Nanogels

High biocompatibility, flexible drug release, and capacity to shield therapeutic materials (proteins or nucleic acids) from deterioration are some of the benefits of nanogels in biomedical applications. Their small size allows them to quickly pass through biological barriers and administer drugs in a targeted and sustained release, minimizing unwanted effects [8,9]. Nanogels can be stimuli-responsive; drug release from these nanogels can be regulated with response to changes in the environment, such as pH, temperature, and redox conditions. Their potential toxicity is a significant drawback, particularly when nonbiodegradable polymers are utilized, as these might accumulate in tissues and have negative consequences [1,10]. The advantages and shortcomings of nanogels in biomedical applications are mentioned in Table 1.

## 4. Advantages of Biodegradable Nanogels

Biodegradable nanogels are at the forefront of nanomedicine because of their adaptability to environmental stimuli, biocompatibility, and adjustable characteristics [49]. Biodegradable polymers are divided into two categories as naturally occurring biodegradable polymers and synthesized biodegradable polymers [50]. Naturally occurring biodegradable polymers include hyaluronic acid [51], alginate [52], heparin [53], chondroitin sulfate [54], chitosan [55], pullulan [56], dextran [57], and gelatin [58] and their potential immunogenic reactions, degradability, drug release behavior, and unregulated structure restrict their use in biomedicine [59,60]. Synthetic polymers with well-controlled structures, such as polyesters, polypeptides, and polyphosphates, can be used to create various nanogels with regulated stability, drug release, and degradability. However, biological cues are absent from synthetic polymers [61,62]. Because biodegradable materials may safely break down in the body into nontoxic byproducts, these materials are ideal for biomedical applications like tissue engineering, gene therapy, medication administration, and immunization [63]. Therapeutic substances can be encapsulated in biodegradable nanogels and released at a regulated rate. This is particularly helpful for inflammatory disease (localized treatment to avoid systemic side effects) and cancer therapy (targeting tumor cells while minimizing systemic toxicity). In reductive intracellular settings, doxorubicin can be delivered to cancer cells specifically using poly(N-isopropylacrylamide)-based nanogels crosslinked with biodegradable disulfide linkages [63]. Biodegradable nanogels promote cellular absorption and shield nucleic acids from enzyme destruction. Because they are biodegradable, they can be released inside cells and then safely degraded [50]. Biodegradable nanogels can also serve as a scaffold for cell adhesion and proliferation by imitating the extracellular matrix. This aids in wound healing and regenerative medicine [64].

## 5. Nanogels for Bioimaging

The combination of biosensing and bioimaging properties of stimuli-responsive nanogels with imaging contrast agents and controlled drug delivery function has been the subject of extensive research [65,66,67]. These responsive hybrid nanogels possess two essential characteristics. To figure out the complex biological processes and create new diagnoses, one is the endogenous and exogenous probing capacity that allows the ongoing monitoring of the biochemical and biophysical parameters over time and place [68,69,70]. Another is the implementation of responsive drug delivery with simultaneous bioimaging, which offers significant benefits over conventional chemotherapy since the imaging function can be used to accurately determine the size and location of the tumor site and track the effectiveness of chemotherapy [71,72,73]. QD-containing polymeric nanogels were employed as biological dyes for live cells or in vivo imaging because they produced a particular light or fluorescence signal. Multi-responsive biosensing nanoparticles were made by combining optical properties of cadmium–selenium (CdSe) QDs with the pH-responsiveness of modified chitosan, protected by chitosan-poly (methacrylic acid) [67]. As a result, the pH range of 5.0 to 7.4 was the ideal range for the regulated release of loaded drug, and optical activities of CdSe QDs allowed for the visual observation of the internalization of the nanogels. Thermo-responsive copolymers were employed as a sharp contrast magnetic resonance imaging (MRI) diagnostic tool [74]. After fabricating a thermo-responsive poly ((N-2,2-difluoroethyl) acrylamide) (pDFEA) and two block co-polymers, PHPMA or PMeOx, the materials self-assembled into nanoparticles at body temperature. Fluorine atoms were then encapsulated in the nanogels as a high-sensitivity MRI probe. Numerous targeted diagnostic nanogel platforms have been assessed for their use in biomedicine; however, the toxicity of those that hold metals is still a problem. To lessen the cytotoxicity, several core–shell structured nanogels were used, preventing the metals from contacting the cell surface. To treat cancer with radiotherapy, chitin–MnO_2_ ternary composite nanogels (ACM-TNGs) based on gold nanoparticles (AuNPs) were created [75]. This system’s purpose was to mitigate the toxicity of MnO_2_ nanorods that absorbed radio waves with low frequencies. The AuNPs and MnO_2_ nanorods were encased in chitin nanogels, a biocompatible shell, to reduce the metal toxicity.

**Figure 2:**
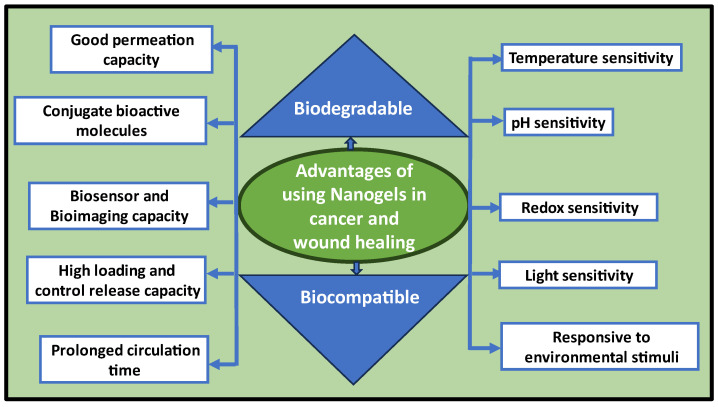
Advantages of using nanogels in cancer diagnostics/therapeutics and wound healing.

A cytotoxicity investigation with L929, HDF, MG63, T47D, and A375 cell lines showed that ACM-TNGs were cyto-compatible and internalizing ACM-TNGs did not change the shape of the cells. A radio frequency of 100 watts per minute was also used to kill breast cancer cells using this nano-system. The nanogels potential as a cell tracker was examined through drug release triggered by NIR light [76]. Another study used ultra-small galodium–gelatin nanogels as an MRI contrast agent because coated gelatin shell lessens the chance that the nanoparticles will pass through the blood–brain and blood–cerebrospinal fluid barriers [77]. To target tumor cells that have elevated levels of folate receptor expression, Jia et al. developed folate-terminated poly (ethylene glycol)-modified hyaluronic acid crosslinked with carbon dots [78]. Tools, gadgets, or systems that combine therapeutic and diagnostic capabilities are referred to as “theranostic”. These platforms were created as part of a drug delivery system to minimize adverse effects, increase therapeutic potential, and diagnose or signal the drug’s biodistribution after administration. Researchers employed PNIPAM-co-acrylic acid with embedded Au nanoparticles, a pH-responsive polymer, as an imaging probe [67]. The protonation of their acrylic acid moieties caused the nanogels to behave in a pH-sensitive manner, while the temperature-sensitive hydrophobic NIPAM groups enhanced their hydrophobic drug loading capability. In another study, hyaluronic acid changed by folate-terminated poly (ethylene glycol) and crosslinked with carbon dots was created to target tumor cells that have elevated levels of folate receptor expression. This system released drugs that were sensitive to pH and acidity, which should be unique to the conditions of tumor tissue. The carbon dots might also be employed for bioimaging [79]. Theranostic properties of a graphene-DOX-entrapped hyaluronic acid nanogel were assessed. When the nanogels were exposed to red-light radiation, the entrapped graphene released light, which is beneficial for imaging cancer cells. CD44, hyaluronic acid receptors, and DOX-targeted release via pH/red-light responsiveness were all highly expressed in cultured non-small lung cancer A549 cells. The nanogels specificity improved the precision of cancer identification while reducing the negative effects [80]. For theranostic applications, another research team developed innovative hybrid nanogels made of superparamagnetic iron oxide nanoparticles (SPIONs), an MRI probe, covered by an alginate shield modified by disulfide. The ability of the nanogels to function as an imaging agent in reductive and acidic environments proved helpful for tumor-targeted drug release and diagnostic targetability. Additionally, a delivery and cell imaging system combining superparamagnetic iron oxide nanoparticles (SPIONs) with a biocompatible alginate derivative showed substantial DOX loading and high toxicity to tumor cells [81]. The schematic representation (Figure 2) mentions various advantages of nanogel in cancer therapy, cancer diagnostics, and wound healing.

## 6. Nanogels as Carrier for Drug Delivery

**Figure 3:**
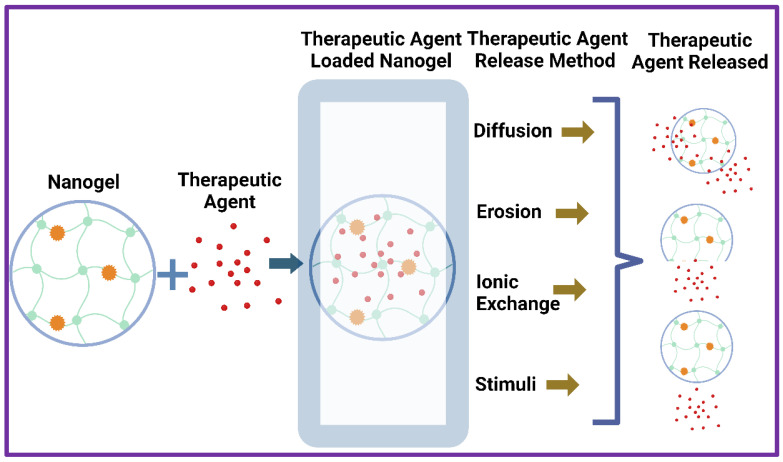
Graphical representation of different methods of drug release from nanogels.

Nanogels are biocompatible, highly stable, and functionally flexible. These characteristics can make using nanogels for delivering drugs easier [82,83]. Nanogels have been utilized extensively as drugs carriers because of their ability to hold drugs with unique properties. The mode of action of the drug is taken into consideration while choosing monomers for nanogels intended for drug delivery [84,85]. Small molecules can be embedded in nanogels because of the pores in their three-dimensional network structure. Nanogels can regulate drug release, mask loaded substances odor, enhance therapeutic efficacy, and minimize undesirable drug reactions [85,86]. The primary methods by which drug released from nanogels are as follows: (1) diffusion; (2) erosion of the nanogels matrix; (3) ionic exchange with the environment; or (4) sensitivity to stimuli like pH, temperature, magnetic field, light, and redox response (Figure 3).

### 6.1. Diffusion

Diffusion is one of the most straightforward methods of drug release from nanogels, and it has been utilized by numerous nanomedicines. The drug diffuses out of the gel due to differences in concentration with the surrounding environment. The substance diffuses from a higher concentration inside the gel to a lower concentration outside the gel [87,88].

### 6.2. Erosion of the Nanogels Matrices

Drugs that have been encapsulated can also be made to release through a process called nanogel breakdown. As nanogels can be biodegradable, their toxicity is lower and unintended accumulation is eliminated during repeated administration. It is possible to provide readily cleavable connections for the polymer backbone. The breakdown may be brought on by specific reducing agents, pH, or even enzymatic activity. Hydrophobic contact encapsulation has been shown to slow down the rate of drug degradation [87,88].

### 6.3. Ionic Exchange with the Environment

Another way to release the drugs is by displacement of counter ions. When a drug-containing cationic nanogel interacts with negatively charged particles on the cell surface or in the surrounding environment, the drug is exchanged for the negatively charged particle [89].

### 6.4. Stimuli Responsiveness

Stimuli-responsive nanogels react to certain internal (pH, redox potential, or enzymes) or external factors (temperature, light, or magnetic fields) by changing structurally or chemically, resulting in the release of drugs [90]. These modifications may cause the nanogel matrix crosslinking linkages to expand, de-swell, degrade, or cleave, which would allow the encapsulated drugs to release gradually at specific locations. For example, redox-sensitive nanogels use high intracellular glutathione levels to sever disulfide bonds and release the payload, whereas pH-sensitive nanogels expand or break down in acidic tumor microenvironments [91]. Likewise, drug release can be triggered by external stimuli such as magnetic fields or near-infrared (NIR) light through localized heating or photothermal effects. This unique release method reduces systemic toxicity while increasing medicinal efficacy [92].

#### 6.4.1. pH-Sensitive Release

Polymers having ionizable groups, including amines and carboxylic acids, makes creating pH-sensitive nanogels easier. Most pH-sensitive interactions between the ionizable groups can be used to form nanogels or conjugate with drugs for stable drug loadings [82,93]. Ionomers, or polyelectrolytes, are the most common polymers used in this technique. These polymers are structurally made up of amine functionality or carboxylic acid. These functions become ionized in response to any change in the surrounding pH, which alters the crosslinked structure of nanogels. Further confirmation of its mechanism, based on the elastic property of polymers, comes from thermodynamic analysis of the polymer–solvent mixture and swelling of nanogels in their unionized form. pH-dependent swelling/deswelling properties of the hydrogel network are explained by electrostatic repulsions that result from ionomers becoming ionized when disseminated in an aqueous solution with a specific pH and ionic strength. The nature and characteristics of the polymers (ionic charge, degree of ionization, concentration, pKa value of ionizable group, and its hydrophilic/hydrophobic behavior) as well as the characteristics of the swelling medium (ionic strength, pH, and the kind and charge density of counter ions) can be used to determine how much such polymers swell [82].

#### 6.4.2. Thermo-Sensitive Triggered Release

Temperature triggered hydrogels can alter their size in response to temperature variations since they are composed of thermosensitive polymers. Depending on their low critical solution temperature (LCST), temperature-triggered hydrogels can be divided into two categories: positive responsive and negative responsive. Positive temperature hydrogels expand at higher temperatures and contract at lower ones compared to the lower critical temperature. At temperatures below LCST, hydrogels with negative temperature exhibit swelling and, at temperatures above LCST, they shrink [94,95]. The range of temperatures at which nanogels can change their volume is known as the volume phase transition temperature (VPTT). Usually, volume variation is used to control drug release and enhance encapsulation. The release of thermosensitive nanogels is caused by one of three mechanisms: simple diffusion, swelling, or degradation. Depending on the chemical composition of the nanogels, the release may or may not cause a burst [96,97].

#### 6.4.3. Magnetic Field-Responsive Release

Iron oxide nanoparticles and polymers are combined to create nanogels that are precisely engineered to respond to magnetic fields. Because of their superparamagnetic and ferro- and ferrimagnetic properties, nanoparticles are most often used in drug delivery [98,99]. By using the emulsion polymerization technique, harmless and biocompatible iron oxide nanoparticles are entrapped in nanogels for precise delivering of pharmaceuticals. Magnetic field sensitive nanogels allow for remote management of the drug distribution [98,100,101].

#### 6.4.4. Photo-Sensitive Release

Polymers with photoactive groups, including azobenzene, spirobenzopyran, or cinnamonyl, are used to create light-sensitive nanogels. These polymers double bonds change from trans to cis when exposed to light, altering the nanogel size and shape [102,103]. Hybrid nanogels are a different class of light-sensitive nanogels that blend noble metals with polymers. In this instance, metals transform light energy into heat, resulting in alteration of the polymer structure. Photosensitive nanogels can undergo cis trans isomerization upon exposure to specific radiations and can expand or contract, responding to temperature changes, hence easing the release of pharmaceuticals [104,105].

#### 6.4.5. Redox-Responsive Release

Nicotinamide adenine dinucleotide phosphate (NADPH) and glutathione (GSH) are reducing chemicals found in cancerous cells, which provide a reducing environment. It is believed that the GSH levels in the tumor microenvironment are four times higher than in normal tissues. This environment eases the fast redox-responsive disintegration of nanocarrier systems, mostly by the reduction of disulfide bonds [106]. Nanogel disintegration releases anticancer drugs into the oxidative state of the tumor. Additionally, the release mostly occurs in the cytoplasm of the target cells, which improves the therapeutic advantages of anticancer drugs [107,108,109,110].

## 7. Nanogels as Carrier of Drug Delivery for Cancer Therapy

One of the primary disadvantages of conventional chemotherapy is its nonselective method, which kills both cancerous and noncancerous cells. It has been effectively shown that stimuli-sensitive nanogels can deliver chemotherapeutic drugs to cancer cells with lower toxicity and fewer side effects [111]. Hormone therapy has been associated with increased risk factors for diabetes mellitus and blood clots. Such risk factors are not associated with nanogels designed for targeted drug delivery in specific cancer types. Chitin-polymerized doxorubicin-loaded nanogels have shown effectiveness in treatment of lung, liver, breast, and prostate cancer [112,113,114]. While many traditional cancer therapies have been improved by cancer immunotherapy. Cancer immune therapy is limited by several physical obstacles and metabolic considerations that do not apply to the use of nanogels [115]. Moreover, immunotherapy for cancer may make noncancerous cells a greater risk. To deliver the ideal numbers of chimeric antigen receptor (CAR) T-cells for immunotherapy, researchers have been successful in creating protein nanogels. Optimized amounts of CAR T-cells were released into the tumor microenvironment by the nanogels in response to T-cell receptor (TCR) activation. Protein release was controlled to guarantee a substantial release of drugs cargo, enhancing efficacy without raising toxicity [116]. Angiogenesis inhibitors are effective in stopping the growth of certain conditions that create new blood vessels. But if cancer prevails over blood supply, inhibitor could not be effective. This restriction of angiogenesis inhibitors is alleviated by the effective tumor-targeting capability of nanogels [117]. This drawback of angiogenesis inhibitors is resolved by the effective tumor-targeting capabilities of nanogels. For tumor targeting, researchers created a poly (N-isopropyl acrylamide co-acrylic acid) nanogel that is pH- and thermo-responsive [118]. Non-selectivity and consequent cell damage are two limitations that radiation treatment and most conventional cancer therapies share. By assuring targeted drug delivery, nanogels not only minimize cell toxicity but can also be designed to combine the advantages of two or more traditional cancer therapies [119]. In a pioneering work, a group of scientists proposed a pH-sensitive and biodegradable nanogel system as a drug nano-carrier for the combination of radiation and chemotherapy. Carboxymethyl cellulose and bovine serum albumin were compounded to create this homogeneously stable nanogel [120]. A hybrid nanogel was effectively loaded with the radionuclide ^131^I and camptothecin, showing a high drug loading capacity of 16.72 by weight percent, a pH-controlled drug release profile, good biocompatibility, and little hemolysis. This combination promoted drug uptake by cells, extended blood circulation, and increased drug accumulation at the tumor location [121]. Fe_3_O_4_ nanoparticles coated with pH and temperature-sensitive poly (N-isopropyl acrylamide co-acrylic acid) gel and transporting citric acid were combined with lactoferrin labeled with Cy 5.5 to serve as a dual-purpose contrast agent for MRI and intraoperative optical imaging for gliomas [122]. According to reports, a thermo-sensitive hydrogel co-loaded with DOX/IL-2/IFN-γ improved B16F10 melanoma tumor response to treatment by enhancing tumor cell death and boosting CD3+/CD4+T and CD3+/CD8+T cell proliferation [111]. Details of nanogels with different therapeutic agents used for cancer drug delivery are mentioned in Table 2.

### 7.1. Nanogel Formulation for Breast Cancer

One of the defining characteristics of cancer microenvironments is hypoxia. It also reduces chemotherapeutic drug effectiveness. Developing hypoxia-responsive nanogels is an innovative method to transform a perceived drawback into a strength. A few anticancer drugs have been changed into nanogel formulations that respond to hypoxia. Although Ribonuclease A (RNase), one of these anticancer drugs, has been shown to have good efficacy and selectivity, it is unstable, has a short half-life, and has low membrane penetration [140]. A hypoxia-responsive Ribonuclease A (RNase) nanogel formulated by researchers has been shown to increase membrane penetration and stability. Azobenzene and β-cyclodextrin coupled to poly (L-glutamic acid)-graft-poly (ethylene glycol) methyl ether (PLG-g-mPEG) were used to create nanogels through host–guest interactions. In mild aqueous conditions, the RNase was loaded into the nanogels. It has been reported that, when the cancer microenvironment was hypoxic, the nanogel released 75% of the RNase [140].

Another group developed dual-targeted nanogels for protein therapy [141]. To improve the targeted delivery of protein therapy for metastatic 4T1 breast cancer in vivo, “epidermal growth factor receptor (EGFR) and CD44 dual-targeted hyaluronic acid nanogels (EGFR/CD44-NGs)” were formulated. Comparison of dual-targeted nanogels to the mono-targeted nanogels showed that protein cellular absorption was more than six times higher. As per findings, dual-targeted protein therapy is a successful treatment for the metastases of breast cancer [141].

### 7.2. Nanogel Formulation for Skin Cancer

The most aggressive kind of skin cancer is melanoma. The physiological makeup of skin, low selectivity, and poor efficacy are obstacles to topical chemotherapeutic drug administration [142,143]. To combat melanoma, researchers formulated a pH-responsive biodegradable nanogel (FCNGL) based on chitosan containing 5-fluorouracil (5-FU). To achieve targeted drug release at the tumor location, the nanogels were engineered to release drug in the slightly acidic cancer microenvironment. Comparing the nanogels to other traditional melanoma formulations, the skin layer integrity was effectively preserved [143].

### 7.3. Nanogel Formulation for Colorectal Cancer

A derivative of camptothecin, irinotecan, targets topoisomerase 1. Irinotecan is still highly effective and important in the treatment of solid tumors, including colorectal cancer, even though it was initially authorized for use as a cancer treatment in Japan 25 years ago [144]. A research group developed gelatin nanogels filled with irinotecan and platelet membrane camouflaged for in vivo treatment of colorectal cancer. The outer cores were made of platelet membranes (PTM), and the inner cores were made of gelatin nanogels (GN) filled with irinotecan (IRN). The entire PTM/GN/IRN complex avoided being cleared by the RES because the outer core was made of a natural membrane. Because the platelet also had the ability to aggregate at tumor sites, the combination was assured to do so [145].

### 7.4. Nanogel Formulation for Prostate Cancer

Short single-stranded DNA or RNA oligonucleotides known as aptamers have high affinity and selectivity for binding with small molecule ligands or protein targets [146]. By conjugating to small interfering RNAs (siRNAs), drugs molecules, or nanoparticles, aptamers (Apt) can effectively transfer proteins, drugs, or nucleic acids into cells along with minimizing side effects [147,148]. DNA aptamer-linked myristate–chitosan nanogels were formulated by Atabi et al. [149] for targeted prostate cancer treatment. LNCap, a selective ssDNA aptamer that can identify androgen-sensitive human prostate cancer cells, was connected to myristate–chitosan nanogels (MCS). Doxorubicin (DOX) was added to the resultant nanogel combination to create the Apt-MCSDOX complex, which targets prostate cancer cells with drug delivery. The Apt-MCSDOX combination was used to treat LNCaP cells. The LNCaP cells were cytotoxically affected by the Apt-MCSDOX combination. This finding suggested that aptamer-based nanogel demonstrated a targeted effect against prostate cancer [149].

### 7.5. Nanogel Formulation for Lung Cancer

The first-line treatment for lung cancer and other solid tumor types is cisplatin. However, cisplatin resistance presents a problem for cancer treatment and it also leads to poor treatment outcomes because of limited drug activity and apoptotic induction [150,151]. To improve cisplatin-induced apoptosis, a multifunctional Valproate-D-nanogel was developed. In human lung adenocarcinoma cancer, the nanogel effectively reversed cisplatin resistance, as shown by a resistance reversal index of 50.22. It was found that the nanogel could successfully prevent resistance to cisplatin [151].

A secondary ginsenoside bio-transformed from major ginsenosides, ginsenoside compound K (CK), can be an anticancer agent. It has a low bioavailability and is not very soluble in water. Because of this, its uses in cancer treatment are restricted [152]. To treat lung cancer, researchers developed pH-responsive nanogels with ginsenoside compound K. Drug release tests were carried out under various pH settings to find the ideal drug release profile. The formulated nanogel showed antitumor efficacy and was 7.7% greater than the free drugs. This proves that CK formulated as nanogels can increase its bioavailability [153].

### 7.6. Nanogel Formulation for Glioma Cancer

Brain and central nervous system cancer, or glioma, is the 17th most prevalent type of cancer. The method by which malignant gliomas form is under study; however, it is thought to be connected to the immune system [152]. It has been established that nanogels are effective at crossing the blood–brain barrier to provide the most tailored treatments for brain cancer [154]. Nanogels functionalized with a diphtheria toxin receptor ligand were created by Singh et al. [155]. This allowed for receptor-mediated transcytosis, or transcellular transport, of the nanogels into the tumor cells across the blood–brain barrier [155]. By employing modified peptide angiopep-2 to create nanogels, Song et al. enhanced doxorubicin ability to penetrate the blood–brain barrier. Teriflunomide (TFM)-loaded intranasal nanogels are a nanogel formulation that is used to assess its anticancer effectiveness [156]. In this nanogel formulation, a range of substances have been employed, such as gellan gum, carbopol 974P, and a mixture of lipids. Nanogel formulations showed anticancer efficacy and were twice as effective as other formulations [157].

### 7.7. Nanogel Formulation for Ovarian Cancer

Using 3D printing technology, developed nanogel discs are filled with rapamycin and paclitaxel as an adjuvant treatment for ovarian cancer. While being stored, the nanogels remained stable. The peritoneal delivery of paclitaxel and rapamycin via nanogel discs was successful in ES-2-luc ovarian cancer-bearing xenograft mice, according to in vivo evaluation [158].

Another group created dendrimer-decorated PVCL-GMA nanogels (NGs) for ovarian cancer treatment by combining poly (amido amine) (PAMAM), poly (N-vinylcaprolactam) (PVCL), and glycidyl methacrylate (GMA) [159]. Dual heat and pH-responsive behaviors were made possible by the polymers special properties that improved targeted drug administration with little side effects [159]. Hyaluronic acid nanogels were created by another researcher as a treatment for ovarian cancer [160]. They created nanogels by chemically crosslinking polyethyleneimine (PEI) and hyaluronic acid (HA) using “a novel emulsion-based strategy”. By improving the physicochemical interactions between HA and PEI, their approach dropped the need for surfactants and produced a stable emulsion. Aqueous solutions of HA and PEI made up the emulsion dispersed phases, while an organic solvent made up the continuous phase. The HA-PEI nanogels were assessed and showed potential for ovarian cancer treatments. When compared to the free drug, they had a superior sustained release profile and were stable [160].

To ensure that ovarian cancer cells are adequately exposed to the best doses of cisplatin, Yamaguchi et al. developed a cisplatin (CDDP)-loaded nanogel for ovarian cancer that is spread peritoneally [161]. They created an alginate (AL)-based hybrid system in which an injectable AL-hydrogel cross-linked with calcium ions encapsulated a CDDP-loaded AL nanogel (AL/CDDP-nanogel). This matrix allowed CDDP to be released from the nanogel hybrid over a week, preventing its quick clearance [161].

## 8. Nanogels as Carrier for Drug Delivery for Skin Dermal and Transdermal

Numerous studies have been conducted on nanogels as effective nanocarriers due to their adaptable physicochemical properties. The nanogels can also be altered to have a certain particle size, shape, and surface charge for improved skin layer penetration. These qualities should be carefully picked though, as they have the potential to trigger the body immune response [162]. Chitosan-based nanogels are extensively documented in the literature; their cutaneous distribution is influenced by the polymer positive charge because of their strong mucin binding [163]. Certain nanocarrier sizes are said to be better suited for transdermal and dermal distribution. For example, in the stratum corneum, the aggregation of rigid nanogels with small (<100 nm) core structures is superior to liposomes because the rigid nanogels may adhere to the intercellular space [164]. Researchers developed and evaluated topically applied chitin nanogels loaded with 5-fluorracil to enhance drug retention in the skin and manage skin cancer. Particles had a mean size of 125–140 nm and a charge of +31.9 mV. The findings showed that the stratum corneum and chitin positive charge nanogel work together to release keratin and ease drug accumulation in the deeper layers of the skin [165]. Furthermore, compared to traditional capsaicin-loaded gels, capsaicin nano emulsion gels (an upgraded version of nanogels) proved superior in skin penetration; this is due to the adjustable size and shape of the nanogel particles [166]. Details of nanogels with different therapeutic agents used for the wound healing process are documented in Table 3. The biopolymers silk fibroin, alginate, chitosan, collagen, and hyaluronic acid are frequently utilized to create nanogels for wound healing applications. These biopolymers have inherent antibacterial and anti-inflammatory properties, which increases their use in wound healing [167,168,169].

One of the major clinical hurdles is scarless wound healing, which raises cosmetic issues. Nanogels can be used to encapsulate growth factors and anti-inflammatory drugs to control cell migration and proliferation, collagen modulators to control remodeling, and angiogenic factors to improve vascularization for the best possible tissue repair. These drugs may have a prolonged release from such encapsulation [192]. For scarless wound healing, a recent study described HA-modified and verteporfin (VP)-loaded polylactic acid nanogel (VP-PLA). Yes-associated protein (YAP) expression was successfully suppressed by the nanogel, which is good because YAP activity is connected to the development of fibrosis. Concurrently, the nanogel increased fibroblast migration rates and stimulated the expression of proliferating cell nuclear antigen (PCNA), a sign of cell proliferation, both of which aid in tissue repair. It is noteworthy that study showed that the PLA component of the nanogel had no direct effect on YAP inhibition, suggesting that the components utilized for the fabrication of the nanogel were adequate to provide the desired outcomes [170]. Paccai eruvai nanogels are another advancement in biomaterials for wound healing and study showed that the nanogel therapy improves wound healing and collagen formation. Compared to the rate attained under typical treatment that may last up to three weeks, the nanogel showed a shorter epithelialization time of up to 14 days, indicating a faster wound healing rate [193].

## 9. Conclusions and Future Prospects

Over time, nanogels have developed into a carrier system that can encapsulate a variety of guest molecules. The development of their synthesis methods and a better comprehension of their material characteristics, such as softness and swelling behavior, are responsible for this. With this knowledge, we may investigate their uses in other biomedical domains and consider how we might improve these characteristics. Advances in analytical methods also help us understand how they behave in vivo, which can guide efforts to enhance their pharmacokinetics, degradation profiles and create future nanogels.

Small molecules such as drugs, fluorophores, proteins, peptides, nucleic acids, and even inorganic nanoparticles made of iron oxide, silver, or gold can be customized to mix well with nanogels.

Depending on the intended use, small carriers can also include a mixture of two or more agents. In recent years, nanogels have evolved into multi-drug carriers and multi-modal imaging agents. Targeting ligands can be presented to a receptor of interest by surface-functionalizing that will bring it to the targeted location. When exposed to external or environmental stimuli, nanogels experience volume phase changes, which cause their crosslinked network to inflate.

This makes it possible to control drug release spatially, temporally and activate reporter molecules, which can produce signals for imaging and diagnostics. Because of these characteristics, nanogels are more applicable than other nanoparticulate systems.

Nanogels used in biomedicine must be biocompatible and should have designs to reduce adverse effects on surrounding tissue and improve the stability and effectiveness of active medications, respectively. Components of core–shell structure of nanogels may be affected by the reactivity of different stimuli (physiological and external environment), protecting the encapsulated drugs and increasing the specificity to the nanocarriers.

Nanogels have the potential to open the door to effective targeted drug delivery to cancerous tissues and cells. Numerous elements, from those connected to the drugs to those related to the patient, interact during cancer chemotherapy. The stability of the dosage form both in vitro and in vivo is crucial to ensure that the dosage reaches the target site undamaged. The major method of delivering cancer drug is intravenously, and nanogels have shown themselves to be a very stable dosage form that can deliver the necessary payload to malignant areas. Topical formulations, however, will be helpful in localized skin malignancies given the adaptability of nanogels, as their permeability can be changed by suitable engineering. Since biomimetic hydrogels are predicted to be biocompatible, circumvent the RES, and alter the nuclear phagocytic system. They are expected to play a key role in intracellular drug administration and result in a longer in vivo circulation period.

The process of wound healing is intricate. In addition to being more expensive and painful for patients, poorly healed wounds—particularly chronic ones—are more challenging to treat.

By releasing drugs or other molecules locally, drug delivery through wound dressings enhances the peri-wound environment, promotes wound healing, and protects the wound while keeping it moist. Using nanotechnology, researchers have created or altered drugs to increase their local availability by boosting their stability, activity, and cell penetration. In addition to ensuring the continuous release of medications in the wound environment, it carried out several local therapeutic effects, including local antibacterial, oxygen delivery, nucleic acid treatment, and scavenging of reactive oxygen species. Based on various theories, researchers have created an expanding number of hydrogel dressings filled with nanomedicine, offering new choices for wound care.

The use of crosslinked nanogels in cancer imaging and treatment has shown a lot of promise in recent years. Nanogels covalently crosslinked nanostructure gives them superior physiological stability, multifunctionality, and drug-release profile when compared to conventional micelles and liposomes. Because of their hydrodynamic size, which typically ranges from tens to hundreds of nanometers, the nanogels can efficiently concentrate in tumor locations. Moreover, nanogels have strong biocompatibility in both in vitro and in vivo settings, suggesting that they may find utilization in clinical translation.

Furthermore, the structure of nanogels can be used to build new therapeutic approaches like EDT, which have a high therapeutic efficacy and little adverse effects. Contrast compounds can be conjugated or placed onto nanogels in addition to medications. These nanogels have been used extensively in cancer treatment that is guided by imaging. While Fe_3_O_4_-coated or Gd-conjugated nanogels are excellent choices for MRI, nanogels loaded with photosensitizers can be employed for photo-theranostics. It may also be possible to create activable chemotherapy-based nano-theranostic systems because of the multifunctionality of nanogels. These crosslinked nanogels have shown many benefits in cancer imaging. Because crosslinked nanogels are more stable than liposomes and micelles, they can stop burst release while in circulation, which enhances drug delivery effectiveness and tumor accumulation.

In most cases, Gd complexes are conjugated onto nanogels for T1-weighted MRI. Nanogels greater physiological stability may allow them to keep their MRI signal in the bloodstream longer than Gd-conjugated micelles.

## Figures and Tables

**Table 1:** Advantages and Shortcomings of Nanogels.

Types of Nanogels	Advantages	Shortcomings
Chitosan-based Nanogel	Cancer: Applied in cancer nanomedicine serving as drug delivery, gene delivery, and bioimaging [35]. Wound Healing: Used to promote wound healing by delivering growth factors and antibacterial agents locally [36]. Antibacterial: Chitosan nanogel loaded with antibacterial drug for infection prevention [37].	The poor colloidal stability of these nanogels is a major disadvantage; they are prone to aggregation, precipitation, and deterioration over time, particularly in aqueous suspensions, which can impair their functionality in biological systems [38].
Polyethylene Glycol (PEG)-based nanogel	Cancer: PEG-based nanogel enhances the stability and solubility of chemotherapy drugs used in cancer treatment [39]	PEG’s nonbiodegradability is a disadvantage since it may accumulate in the body over time and might result in long-term toxicity [40]
Liposome-based nanogel	Cancer: For cancer therapy, Matrix metallopeptidase-2-responsive polypeptide nanogel-coated double-targeted liposomes for chemotherapy and improved immunotherapy against cervical cancer [41]. Gene Delivery: Liposome hydrogel nanoparticles used for the targeted delivery of CRISPR/Cas9-mediated cancer gene therapy [42]	One significant problem is their chemical and physical instability, which can impair the effectiveness of treatment. This includes propensity for phospholipid breakdown, drug leakage, and aggregation during storage [43]
Poly (N-isopropylacrylamide) (PNIPAM)-based nanogel	Cancer: Nanogels made of poly(N-isopropylacrylamide) showed thermosensitive self-assembly and GSH-triggered drug release for effective tumor treatment [44]. Tissue engineering: In cardiac tissue engineering, PNIPAM-based hydrogels have been used to promote the growth and differentiation of heart stromal cells, aiding in the restoration of damage caused by myocardial infarction [45].	The regulated distribution of therapeutic agents may be jeopardized by PNIPAM’s poor drug loading capacity and propensity for instantaneous drug release following temperature changes [45].
Dextron-based nanogel	Cancer: Because dextrin-based nanogels are biocompatible, biodegradable, and responsive to stimuli unique to tumors, they have become intriguing vehicles for targeted cancer therapy [46]. Targeted delivery: Dextron-based nanogel has been used for targeted delivery of siRNA [47].	Long-term toxicity could result from accumulation of degraded products in the body brought on by the breakdown of dextran nanogels [48]

**Table 2:** Nanogels Reported for Drug Delivery Applications in Cancer.

Therapeutic Agents	Nanogels Adopted	Cancer Types	Properties	References
Doxorubicin	^H^PMPC Nanogel	Live cancer	Showing anticancer activity with good biocompatibility both in vitro and in vivo.	[123]
Hyaluronate (HA)	Photolabile 4-(4-(1-hydroxyethyl)-2-methoxy-5-nitrophenoxy)butyric acid (HMNB)	Human nasopharyngeal epidermal carcinoma	Significant improvement in KB tumor-cell-killing efficacy	[124]
Doxorubicin hydrochloride	poly [(N-isopropylacrylamide)-co-(2-dimethylamino ethyl methacrylate) Nanogel	Breast cancer	A significant decrease in toxicity was observed in the case of doxorubicin embedded in hydrogel.	[125]
Doxorubicin	Injectable shear-thinning hydrogel (STH)	Breast cancer and Glioblastoma	Increase overall survival in breast tumor- and glioblastoma-bearing animal models.	[126]
Doxorubicin	Nanohybrid of hyaluronic acid (HA)-decorated graphene oxide (GO)	Hepatic cancer	Higher tumor inhibition rate for mice having H22 hepatic cancer cells when compared to the GO-DOX formulation and free DOX.	[127]
5-fluorouracil (5-FU)	Alginate-modified graphene oxide	Liver cancer	Increased the mice life period and markedly reduced tumor growth and liver metastasis.	[128]
Doxorubicin	Lactobionic acid and carboxymethyl chitosan functionalized graphene oxide nanocomposites	Liver cancer	Target drug delivery to liver cells and efficiently trigger cell death	[129]
miR155	Nucleic acid Nanogel	Glioblastoma	Strong tumor-targeting ability along with outstanding tumor-inhibition effectiveness against glioblastoma.	[130]
siRNA	Polydopamine-coated nucleic acid Nanogel	Human cervical carcinoma	Anticancer activity against cervical cancer tumor induced by Hela cells.	[131]
Doxorubicin	Redox-responsive cisplatin Nanogels	Ovarian cancer	Boost anticancer activity against cisplatin resistance ovarian cancer	[132]
Iodine-labeled RGDY	^125^I-GNR-RGDY hydrogel	Breast cancer	During a 4-week course of NDN hydrogel treatment, the combination of continuous brachytherapy and photothermal effect effectively avoided wound infection and breast cancer recurrence.	[133]
siRNA	Nucleic acid Nanogel	Melanoma	Increases antitumor efficacy in a synergistic manner and significantly boosts the anticancer immune response.	[134]
CRISPR/Cas9	Noncationic nucleic acid Nanogel	Cervical cancer	Increased cellular absorption effectiveness and delivery system ability to modify the target genome.	[135]
IMS/ICG	GSH/ROS dual response Nanogel	Solid tumor	Developed for endorsing cancer immunotherapy	[136]
Gemcitabin, R837	Immunomodulatory multidomain nanogel (iGel)	Triple negative Breast cancer and Cervical cancer	Anticancer efficacies against TNBC and TC1 cervical cancer cells	[137]
Axitinib	Injectable hydrogel		Modulate T cells for immunotherapy against cancer	[138]
Thiocolchicoside and lauric acid	chitosan Nanogel	Oral cancer	Anticancer activity against oral cancer cell lines	[139]

**Table 3:** Application of Nanogels in Wound healing.

Therapeutic Agents	Nanogels Adopted	Wound Types	Properties	References
Hyaluronic acid/verteporfin	Polylactic acid nanogels	Wound re-epithelialization	Promote scarless wound healing by controlling scar formation and speeding up wound re-epithelialization.	[170]
Curcumin	Fish scale collagen-HPMC nanogel	In vivo murine wound model	FSC-HPMC nanogel showed safe, promising, and more stable material for wound healing applications.	[171]
Polyherbal *Mattan tailam*	Novel *Mattan tailam* Nanogel	Rats skin wound	Formulation significantly increases collagen synthesis, tensile strength, and wound contraction.	[172]
Copper sulfide (CuS)	Gelatinase Responsive Nanogel	Mice wound infected with S. aureus	Improved wound healing and removed the colonized microbes from mice *S. aureus* infected wounds.	[173]
Lidocaine	Lidocaine-loaded nanoemulsion convert into gel using carbopol-940 as a gelling agent	In vivo mice wound	The dermatokinetic profile of nanogel was superior than that of traditional gel.	[174]
AgNPs	CLT-AgNPs Nanogel	Acute and chronic wounds	Promotes agranulocytosis and fibroblast growth, resulted efficient and quick wound healing.	[175]
Nitric oxide	Sprayable chitosan Nanogel	Diabetic wound	Accelerate diabetic wound healing through bacteria inhibition, biofilm eradication and macrophage polarization	[176]
Eugenol	Eugenol-loaded nanogel + PCL/Cs nanofiber	Excision wound model in Wistar rats	Promoting wound healing by reducing inflammation and edema and encouraging angiogenesis, collagen synthesis, and re-epithelialization.	[177]
Sacchachitin	Micronized sacchachitin (*m*SC) Nanogel	Superficial chemical corneal burns	Wound healing against corneal epithelium	[178]
Exosomes	Exosome-coated oxygen nanobubble-laden hydrogel	Rat full-thickness wound model	Promoting angiogenesis, boosting exosome distribution, improving healing, lowering hypoxia, and preventing inflammation in a male rat full thickness wound model	[179]
Silver nanoparticles	Ficus lacor-silver nanoparticle gel	Rat excision wound model	Demonstrated considerable improvement in the excision wound model	[180]
Lignin	Lignin-Incorporated Nanogel		Helping as an antioxidant biomaterial for Wound healing	[181]
Silver Sulfadiazine	Nanosuspension-Based *Aloe vera* Gel	Burn wounds in mice	Improved burn wound Healing	[182]
Silver nanoparticles	Neomycin-silver nanocomposite	Rat excision wound model	Potential wound healing activity	[183]
Melatonin	Melatonin Nanogel	Burn wounds of rats	Promoted epidermis growth with evident wound contraction	[184]
Berberine	Hyaluronan/Poly-L-lysine/Berberine Nanogels	In vitro on fibroblast cells	In 42 h, berberine-loaded nanogels were able to fully seal the fibroblast gap	[185]
Gentamicin	Gentamicin nanogel films	Rat full-thickness excisional model	Developed a more effective and affordable topical therapy for the healing of cutaneous wounds.	[186]
Atorvastatin	Atorvastatin-Loaded Nanoemulgel	Ex vivoRat skin model	Enhancing wound healing	[187]
Interleukin-2	Chitosan-Based Nanogel	Rat wound	Enhancing wound healing by raising GSH levels in injured tissues and lowering MDA levels.	[188]
Trinitroglycerin	Chitosan Nanogel	Full-thickness skin wounds model	Continued release of trinitroglycerinfrom nanogel improved wound tissue vascularization	[189]
Chitosan	Chitosan thiocolichoside lauric acid Nanogel	In vitro scratch wound healing	Cytoprotective and wound healing activity	[190]
Vitamin C derivative	Gelatin and Alginate Nanogel	Chronic wound healing	Regulating the inflammatory wound microenvironment.	[191]

## Data Availability

No new data were created or analyzed in this study.
